# Disentangling Risk and Uncertainty: When Risk-Taking Measures Are Not About Risk

**DOI:** 10.3389/fpsyg.2018.02194

**Published:** 2018-11-15

**Authors:** Kristel De Groot, Roy Thurik

**Affiliations:** ^1^Department of Applied Economics, Erasmus School of Economics, Erasmus University Rotterdam, Rotterdam, Netherlands; ^2^Erasmus University Rotterdam Institute for Behaviour and Biology, Erasmus University Rotterdam, Rotterdam, Netherlands; ^3^Institute of Psychology, Erasmus School of Social and Behavioural Sciences, Erasmus University Rotterdam, Rotterdam, Netherlands; ^4^Montpellier Business School and Labex Entreprendre, Montpellier, France

**Keywords:** risk, uncertainty, DOSPERT scale, BART, decision-making

## Abstract

Many studies claim to measure decision-making under risk by employing the Domain-Specific Risk-Taking (DOSPERT) scale, a self-report measure, or the Balloon Analogue Risk Task (BART), a behavioural task. However, these tasks do not measure decision-making under risk but decision-making under uncertainty, a related but distinct concept. The present commentary discusses both the theoretical and empirical basis of the distinction between uncertainty and risk from the viewpoint of several scientific disciplines and reports how many studies wrongfully employ the DOSPERT scale and BART as risk-taking measures. Importantly, we call for proper distinguishing between (tasks measuring) decision-making under uncertainty and decision-making under risk in psychology, and related fields. We believe this is vital as research has shown that people’s attitudes, behaviour, and brain activity differ between both concepts, indicating that confusing the concepts may lead researchers to erroneous conclusions.

## Introduction

Many studies claim to examine decision-making under risk, using a broad range of measures to indicate an individual’s level of risk-taking. Well-known examples of these measures include the Domain-Specific Risk-Taking (DOSPERT, Blais and Weber, 2006) scale, a self-report measure, and the Balloon Analogue Risk Task (BART, Lejuez et al., 2002), a behavioural measure. However, despite including the word ‘risk’ in their names, the DOSPERT scale and BART in fact do not deal with attitude towards risk. The concept they deal with is attitude towards uncertainty, a related but distinct phenomenon that is often contaminated with risk attitude in psychological literature.

## The Theory Behind Decision-Making Under Uncertainty Versus Risk

In economics, the distinction between uncertainty and risk proposed by Knight (1921) has become classic and has been hardly contested. In the case of risk, the outcome is unknown, but the probability distribution governing that outcome is known. Uncertainty, on the other hand, is characterised by both an unknown outcome and an unknown probability distribution. In both cases, preferences are defined across chance distributions of outcomes. For risk, these chances are taken to be objective, whereas for uncertainty, they are subjective. Consider betting with a friend by rolling a die. If one rolls at least a four, one wins 30 Euros (or Pounds, Dollars, Yen, Republic Dataries, Bitcoins, etc.). If one rolls lower, one loses. If the die is unbiased, one’s decision to accept the bet is taken with the knowledge that one has a 50 per cent chance of winning and losing. This situation is characterised by risk. However, if the die has an unknown bias, the situation is characterised by uncertainty. The latter applies to all situations in which one knows that there is a chance of winning and losing but has no information on the exact distribution of these chances.

When laypersons talk about risk, they generally mean uncertainty, as the outcome probabilities are seldom known in everyday situations. In contrast to laypersons, scientists cannot afford to confound the concepts of risk and uncertainty. Contaminating these 2 concepts and hence not adhering to the uncertainty/risk (U/R) distinction is problematic, as this distinction has been supported by various studies showing that it is not only conceptually but also empirically valid. These studies primarily come from three scientific disciplines: economics, psychology, and neurobiology.

## Findings From Economics

Behavioural economic literature has supported the validity of the U/R distinction by showing that individuals are less sensitive to likelihood information in the case of uncertainty compared to risk: likelihood insensitivity decreases with more information (Kahneman and Tversky, 1979; Kahn and Sarin, 1988; Kilka and Weber, 2001; Abdellaoui et al., 2005, 2011; Baillon et al., 2012, 2013, 2017). This phenomenon can be illustrated with the use of a fictional lottery. In the lottery, the difference between a win probability of 0 and 0.1 is substantial, for it reflects the difference between no chance versus a chance. The difference between a probability of 0.9 and 1 is also large, for the latter means a certain win. By contrast, the difference between 0.3 and 0.4, or between 0.6 and 0.7 seems small. If one is not even sure whether these 0.3 and 0.4 (or 0.6, and 0.7) probabilities are accurate (uncertainty), it is plausible that one will treat them as equivalent. Hence, the less information individuals have (uncertainty vs. risk), the more they fail to sufficiently discriminate between different levels of likelihood (Tversky and Kahneman, 1992; Baillon et al., 2012; Baillon, 2015). In addition, the behavioural economic literature shows aversion towards uncertain compared to risky choices (something referred to as ambiguity aversion): individuals prefer known probabilities over unknown probabilities, even if the known probability is low and the unknown probability could be a guaranteed win (Ellsberg, 1961). In the original experiments, this phenomenon is illustrated with urns. Imagine there are 2 urns: the ‘known’ urn, with 50 red and 50 black balls, and the ‘unknown’ urn, which contains 100 balls that are red or black in an unknown proportion. Winning is achieved by drawing a red ball. Which urn do people want to draw from? When asked this question, most people opt for the known urn. However, if they win when drawing a black ball, they also opt for the known urn. This decision contradicts the notion of probability: people act as if the chance of drawing a red ball from the unknown urn is less than 50 per cent, but also as if the chance of drawing a black ball from that same urn is less than 50 per cent. This so-called Ellsberg paradox illustrates our initial statement: when asked to choose, individuals prefer risk over uncertainty.

## Findings From Psychology

Next to behavioural economic literature, psychological literature also supports the empirical distinction between uncertainty and risk. Buckert et al. (2014), for example, show that the cortisol response to stress impacts decision-making under risk, but not under uncertainty. In addition, several studies show how risk and uncertainty are differentially impaired in a broad range of (neuro) psychological disorders. For example, decision-making under uncertainty but not under risk has shown to be impaired in patients who have undergone unilateral temporal lobe surgery (Bonatti et al., 2009), patients with gambling problems (Brevers et al., 2012), breast cancer patients receiving adjuvant chemotherapy (Chen et al., 2013), patients with obsessive-compulsive disorder (Starcke et al., 2009, 2010; Kim et al., 2015; Zhang et al., 2015a,b), and patients with pathological buying issues (Trotzke et al., 2015). The same dissociation holds for normal ageing (Zamarian et al., 2008). The situation, however, is reversed for patients with Parkinson’s disease, who are differentially impaired in decision-making under risk, but not in decision-making under uncertainty (Euteneuer et al., 2009), which could be explained by the notion that decision-making under risk depends more on executive functioning than does decision-making under uncertainty (Brand et al., 2006). These dissociations provide further support for the empirical distinction between uncertainty and risk.

## Findings From Neurobiology

Extending the psychological literature, several studies from the field of neurobiology indicate that risk and uncertainty are differentially coded in the brain. Currently, 2 hypotheses exist outlining how dealing with uncertainty versus risk differs neurobiologically (Schultz et al., 2008). First, both situations may recruit different brain systems, resulting in double dissociations. This hypothesis is supported by studies showing that risk recruits the orbitofrontal cortex, striatum, insula, and (posterior) parietal cortex, whereas uncertainty recruits the amygdala and parts of the frontal cortex such as the inferior frontal gyrus, and the (dorsal) lateral prefrontal cortex (Huettel et al., 2006; Krain et al., 2006; Platt and Huettel, 2008; Schultz et al., 2008; Bach et al., 2009). Second, risk and uncertainty may recruit a common brain mechanism but to different degrees, showing stronger responses to either ambiguous or risky choices. This idea is backed up by studies showing that the activity in some brain structures, such as the orbitofrontal cortex and amygdala, is positively related to the level of uncertainty in a task, whereas activity in the striatal system is negatively correlated (Hsu et al., 2005; Platt and Huettel, 2008; Schultz et al., 2008; Levy et al., 2010). These findings support a graded rather than an all or nothing difference between how uncertainty and risk are neurobiologically coded. There is no conclusive evidence yet on whether uncertainty and risk are mutually exclusive or graded represented in the brain. However, regardless of which hypothesis is supported, uncertainty and risk can be said to differ from each other even at a biological level, which is an important signal of the essential difference between uncertainty and risk.

## The Dospert Scale and Bart

The discussed behavioural economic, psychological, and neurobiological studies demonstrate how uncertainty and risk differ not only on a theoretical basis, but also empirically. This emphasises the need to properly distinguish between the 2 concepts in research. However, as discussed, 2 paradigms that claim to measure decision-making under risk in fact deal with uncertainty: the DOSPERT scale and the BART. For the DOSPERT scale, items include ‘going camping in the wilderness,’ ‘drinking heavily at a social function,’ and ‘investing 10 per cent of your annual income in a new business venture’. For all these items and the others, the outcome distribution is unclear. There is a level of uncertainty, but since the probability distribution of the described situations is not known, this cannot be qualified as risk in the Knightian sense. The same is true for the BART. In the BART, individuals pump up a balloon that can explode at any time. Since the probability distribution of explosions is unknown to the participant (‘participants were given no detailed information about the probability of an explosion,’ Lejuez et al., 2002, p. 77), there is again a level of uncertainty, which cannot be qualified as risk. For the DOSPERT scale, the uncertainty is bi-directional: both the researcher and the participant are ignorant of the probability distribution. For the BART, the uncertainty is one-directional: from the viewpoint of the researcher, the risk is known, as he or she knows how the probability distribution of the task has been programmed. The participant, on the other hand, is not given any information about this.

Although the DOSPERT scale and BART are both clear examples of uncertainty measures, studies applying them do generally not acknowledge that the measures deal with decision-making under uncertainty instead of risk. To examine how pervasive this mistake is, a literature review was conducted using Scopus across November, 2017. The first search parameter concerned the full name of both tasks as mentioned in the article title, abstract, or keywords as indexed in Scopus. This resulted in 17 articles on the DOSPERT scale, and 289 articles on the BART. These 306 articles were all read and classified into 4 categories. An article was categorised as recognising the uncertainty nature of the DOSPERT scale or BART if the article explicitly mentioned that the DOSPERT scale or BART measures decision-making under uncertainty (instead of risk) because the probabilities relevant for the task are unknown to the participant, or if the article seemed to implicitly understand the difference between decision-making under uncertainty and risk, for example by discussing the conceptual difference between known versus unknown probabilities in relation to the experimental paradigms. However, articles were not included in this category if they only stated that probabilities in the task were unknown (which is simply a task characteristic), without relating this to uncertainty or risk. Articles were also not included if they correctly stated that the task measured uncertainty but mentioned invalid reasons for this (for example because ‘the outcome is unknown,’ which is a characteristic of both risk and uncertainty). Articles were classified as not recognising the U/R distinction if they did not seem to be aware of the distinction between the 2 concepts in relation to the DOSPERT scale or BART. This was for example reflected by consistently using the concepts intertwined without discussing the difference; explicitly stating that uncertainty and risk are equal; discussing uncertainty and risk but not relating this to the DOSPERT scale or BART; or stating that the DOSPERT scale or BART measures decision-making under risk, while not mentioning uncertainty (or related concepts such as ambiguity, or unknown probabilities) at all. The 2 remaining categories concerned ‘not accessible’ (if the full text of an article could not be accessed) and ‘not applicable’ (if the article did not say anything concerning either uncertainty or risk, for instance because the DOSPERT scale or BART was used for measuring a different construct, such as impulsivity). The second search parameter concerned the abbreviation of both measures, again as mentioned in the article title, abstract, or keywords as indexed in Scopus. This resulted in 45 articles on the DOSPERT scale, and 3137 on the BART. For these articles, the abstracts were examined, and irrelevant articles (e.g., articles discussing Bart Syndrome) were removed from the search findings. In addition, articles that were already included based on the first search parameters were also removed. The remaining articles were again all read and classified according to the abovementioned criteria. The final categorisation consisted of 48 articles on the DOSPERT scale, and 302 articles on the BART. The included articles were solely identified via the search parameters; no additional method of including articles was employed.

**FIGURE 1:**
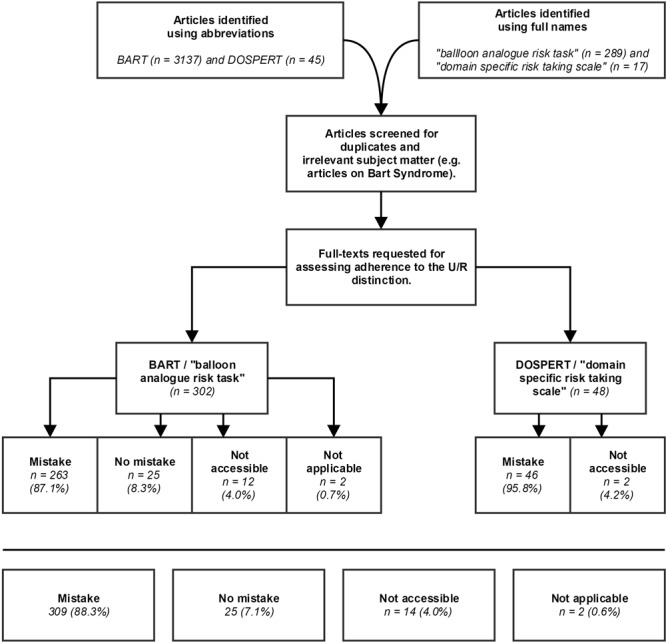
Summary of the categorization process and outcomes.

The results from the categorisation can be found in Figure 1. The findings were in line with our proposition that most studies do not adhere to the U/R distinction and do not correctly identify the DOSPERT scale and BART as uncertainty measures. This was also true for several studies published in this journal. Overall in the literature, only 7.1 per cent of articles correctly adhered to the U/R distinction in relation to the DOSPERT scale and BART. The Knightian distinction between uncertainty and risk was not adhered to by 88.3 per cent of articles. The classification of all examined articles was archived in an online repository and can be accessed via a weblink that is available from the authors upon request. In addition to the basic classification, the repository holds information indicating whether the authors thought a certain classificatory decision was up for debate. This was the case for 12.3 per cent of articles. For these articles, the arguments on which the final decision was based are also reported. The most common arguments reported are discussing the U/R distinction in relation to other tasks but not to the DOSPERT scale or BART; correctly stating that the DOSPERT scale or BART measures decision-making under uncertainty but reporting invalid arguments for this claim; and applying a Bayesian learning paradigm to the BART that quantifies the decision maker’s changing uncertainty about the chances of the balloon exploding, but that does not explicitly say anything regarding the U/R distinction. All articles characterised by these arguments were categorised as not adhering to the U/R distinction, which resulted in a relatively conservative classification. However, the percentage of articles not properly adhering to the U/R distinction in relation to the DOSPERT scale and BART remains high (namely 78.3 per cent) if all articles now classified as ‘mistake’ + ‘up for debate’ were classified as properly adhering to the distinction.

## The Importance of Distinguishing Between Decision-Making Under Uncertainty Versus Risk

From the literature review, we conclude that not properly adhering to the U/R distinction is a widespread problem. Most articles do not even mention the distinction, let alone correctly identify the DOSPERT scale and BART as measuring decision-making under uncertainty instead of risk. However, the present finding that 88.3 per cent of articles does not adhere to the Knightian distinction between uncertainty and risk in relation to the DOSPERT scale or BART does not necessarily mean that 88.3 per cent of authors are unaware of the distinction. In fact, we believe that most researchers understand the conceptual distinction between uncertainty and risk, but do not explicitly report on this in their articles. This absence of uncertainty/risk information in articles could simply be the result of common practises within the field. In psychology, terms such as ‘risk-taking’ and ‘riskiness’ often signify not only a known chance but also a directional effect: a high chance of loss. Uncertainty does not allow for a similar directional connotation, which may result in using the term less frequently. Furthermore, if only few studies explicitly distinguish between uncertainty and risk, this becomes the default within a field, leading others to also not report on this distinction even though they may have considered it when designing their study. This is reflected in the observation that articles that adhere to the U/R distinction are not only scarce but are also not consistently referred to in the literature. This contributes to the contamination of both concepts that currently dominates the literature, and makes research prone to confusion, especially when crossing disciplinary lines.

Being aware of the distinction between uncertainty and risk and applying this knowledge in scientific writings not only is of great importance for scientific coherence but also has meaningful practical implications for government and business because the rules used for decision-making under risk differ from those used for decision-making under uncertainty. As an example, Angner (2012) discusses the regulation of new and unstudied chemical substances. There is little hard data on them, but there is some probability that they will turn out to be toxic. If a policy maker would argue that the decision at hand concerns uncertainty, he or she would have to decide that the new chemical should be banned or heavily regulated until its safety can be established. Speaking in behavioural economic terms, either the minimax (minimising the maximum amount of deaths) or the maximin (maximising the minimum amount of profit) criterion applies in this situation. However, if the policy maker argues that one can and must assign probabilities to all outcomes, he or she faces a choice under risk, and will probably permit the use of the new chemical because the probability that it will turn out to be truly dangerous is low (the expected utility, the alternative with the greatest amount of utility in the long run, is highest for permitting the use). This example shows that decision-making under uncertainty versus risk results in different responses. Therefore, whether a decision is treated as a choice under uncertainty or under risk can have real consequences.

## What Should Researchers Do?

The aim of the present commentary is not to scold researchers from fields such as psychology for not using terminology and conventions used in economics. We do, however, encourage researchers to properly distinguish between uncertainty and risk. We believe that the majority of researchers are in fact already aware of this distinction, even though this is not always reflected in their writings. Moreover, the aim of our commentary is not to take credit for the idea that the DOSPERT scale and BART do not measure attitude towards risk but rather towards uncertainty. In fact, we mention several previous studies that explicitly contribute to this view by providing empirical support for the conceptual distinction. Furthermore, studies adhering to the U/R distinction in relation to the DOSPERT scale or BART can be found in the repository.

The aim of the present commentary is to unite previous research, and to make researchers explicitly aware of the distinction between uncertainty and risk. In addition, the aim is to advise researchers on what tasks (not) to use. For example, a self-report measure probing pure risk-taking should include clear indicators of the probability distribution underlying the outcomes of the described activities. Furthermore, the BART should not be used for measuring pure risk-taking. Instead, different behavioural tasks such as the Cambridge Gambling Task (CGT, Rogers et al., 1999), Game of Dice Task (GDT, Brand et al., 2005), and Columbia Card Task (CCT, Figner et al., 2009) should be used when aiming to measure decision-making under risk. It should be noted though that in real life the chances are almost always unknown, which means that risk has more theoretical than practical importance. Therefore, there is certainly merit to using the DOSPERT scale and BART, especially considering their good external validity. In fact, we could even speculate that this good external validity can be explained by the fact that these tasks measure decision-making under uncertainty (and not risk), which corresponds well to the structure of decision-making in real life. Looking even closer at how decision-making in real life is accomplished, it appears that the distinction between uncertainty and risk is continuous rather than binary. In many cases, individuals have some estimate of the involved probabilities, which develops as they move further along in the decision-making process and receive feedback by sampling the environment. This development is mirrored, for example, in the Iowa Gambling Task (IGT, Bechara et al., 1994), in which participants learn the probabilities associated with card decks as they progress through the task. It could even be argued that the BART is characterised by a learning process as well, which is reflected by studies applying learning models to the task. However, regardless of what (version of a) task is used, it is important to be explicitly aware of what it is measuring: decision-making under uncertainty (BART and DOSPERT scale), decision-making under risk (CGT, GDT, and CCT), or a gradual shift from decision-making under uncertainty to decision-making under risk (IGT and possibly BART). This way, the used nomenclature can stay pure and help readers identify what concepts are examined in a particular study.

Scientists are expected to outperform laypersons in properly distinguishing between concepts. Considering the fluidity of interdisciplinary research, it is pertinent to employ a sole and clear-cut definition of concepts across fields. This is particularly important if concepts have been shown to differ both theoretically and empirically. The present commentary calls for distinguishing uncertainty and risk in the field of psychology and related fields where decision-making under uncertainty and decision-making under risk play an important role, such as neuroeconomics. This will help in using tasks that actually measure the concept one is interested in measuring, which will certainly aid in finding true relationships.

## Author Contributions

KDG conceived the study, performed the literature review, and wrote down the background of the review and its results. RT participated in the design of the search and revised the manuscript critically for important intellectual content. Both authors read and approved the final manuscript.

## Conflict of Interest Statement

The authors declare that the research was conducted in the absence of any commercial or financial relationships that could be construed as a potential conflict of interest.
